# The relationship between psychological abuse, athlete satisfaction, eating disorder and self-harm indicators in elite athletes

**DOI:** 10.3389/fspor.2024.1406775

**Published:** 2025-01-10

**Authors:** Erin Willson, Stephanie Buono, Gretchen Kerr, Ashley Stirling

**Affiliations:** Faculty of Kinesiology and Physical Education, University of Toronto, Toronto, ON, Canada

**Keywords:** emotional abuse, abuse in sport, mental health, disordered eating, safe sport, non-suicidal self-harm, aesthetic sports

## Abstract

**Introduction:**

Psychological abuse continues to be the most frequently reported type of maltreatment among athletes leading to negative mental health such as low mood and self-esteem, increased anxiety, self-harm, and eating disorders. Preliminary evidence suggests athlete satisfaction can influence the perceived outcomes associated with psychological abuse. Despite its negative impacts on athletes, psychological abuse continues to be justified as a tool to enhance athletic performance.

**Methods:**

This study sought to examine the effects of psychological abuse on athlete satisfaction and mental health indicators of eating disorders and self-harm. Participants consisted of Canadian National Team athletes who completed a survey of maltreatment in sport with items assessing psychological abuse, athlete satisfaction, eating disorder and self harm indicators (*n* = 794).

**Results:**

Results indicated a negative correlation between psychological abuse and athlete satisfaction, and a positive correlation with eating disorders and self-harm indicators. Additionally, athlete satisfaction was a significant moderator of psychological abuse on eating disorder and self-harm indicators. High athlete satisfaction buffered against the negative effects of psychological abuse on self-harming indicators in non-aesthetic and weight based sport and non-team sport athletes. Conversely, the interaction between athlete satisfaction and psychological abuse was marginally significant in predicting increased negative effects on eating disorders in aesthetic and weight based sport athletes.

**Discussion:**

These findings highlight the detrimental effects psychological abuse can have on athletes in relation to eating disorders and self-harm, and the buffering role of athlete satisfaction on self-harm but not eating disorder indicators in aesthetic and weight based sport athletes. Recommendations include increased attention to preventing psychological abuse in sport.

## 1 Introduction

Psychological abuse has consistently been identified as the most prevalent form of maltreatment among athletes (1–5), encompassing behaviors such as yelling, swearing, belittling, public humiliation, and negative comments about one's body (6–9). Prevalence studies have indicated psychological abuse can be perpetrated by coaches, peers or teammates, trainers and others (5, 10). While psychological abuse is increasingly acknowledged and explored by researchers, empirical evidence on the impacts of this type of harm on athletes is in a nascent stage. Psychological abuse has been found to have negative impacts on mental health and well-being, particularly self-harm and eating disorder indicators (9, 11–14).

Broadly, eating disorders are defined as persistent disturbances in eating patterns and distressing thoughts or emotions related to food, body shape, and/or weight (15). Several researchers have demonstrated the links between psychological abuse in sport and eating disorders (1, 16, 17). Athletes who experience psychological abuse have reported eating disorder-related behaviors, such as excessive exercise, food and water restriction, obsessive weight/body preoccupation, and binging and purging (9, 12, 18). McMahon and McGannon (18) suggest these behaviors are potentially used to reconfigure and manage endured trauma. At this time, minimal quantitative data exist linking psychological abuse with eating disorder outcomes in athletes.

Self-harm has also been identified as a potential outcome of psychological abuse in sport (12, 13). Self-harming indicators can include ideation or attempts of suicide and non-suicidal self-injury (e.g., burning, cutting, scratching (19). Self-harm has been reported as a common outcome of psychological abuse outside of sport, particularly in women and children (20, 21). Less attention has been given to this relationship in sport, however, preliminary evidence suggests a link. For instance, in a qualitative study on the effects of emotional abuse, Kerr and colleagues (12, p. 84) shared an athlete's reported suicide ideation: “I locked myself in the bathroom and like, I took a bath… and that's about the time I had really really hard ideas, like if I took my own life right now, like who would notice?” Additionally, Daignault and colleagues (11) reported athletes who experienced psychological harm were more likely to report non-suicidal self-injury. Further study of the relationships between psychological abuse and self-harm outcomes is warranted.

Reports of eating disorders and self-harm vary based on sex and sport. Eating disorders have been reported more frequently among women compared to men, in aesthetics and weight-based sports, and in individual sports (22–25). Suicidal ideation and attempts were more common among male athletes and athletes who played football and basketball (i.e., team sports), but also occurred in individual sports like swimming and track and field (26). A more recent study reported the highest rates of suicide in athletes occurred in shooting and fencing, which are both individual sports (27). While these findings provide some insight into the rates of eating disorders and self-harm in sport, there is still minimal investigation of the effects of psychological abuse on eating disorders and self-harm among different sexes and sport types (e.g., team and individual sports). As such, more work is needed to explore potential outcomes of psychological abuse across various populations.

The relationship between psychological abuse and performance is seemingly paradoxical. While some athletes and coaches have claimed these behaviors improve performance, other researchers have demonstrated psychological abuse can decrease satisfaction, motivation, and a desire to participate in sport (12, 28). Kerr and colleagues (12, p. 64) studied the effects of emotional abuse, revealing both positive and negative impacts; for example, an athlete suggested it helped them work harder but also negatively impacted their mental health: “I will say that having a coach that was a bit of a bully didn't do me any favors. but in the same breath…that might have made me work harder”. Stirling and Kerr (28) also investigated the effects of performance satisfaction, a facet of overall athlete satisfaction, or a “positive affective state” in relation to an athlete's experiences (29). Results demonstrated that when performance satisfaction is high, emotionally abusive practices were interpreted more positively. On the other hand, when performance satisfaction decreased, there was an increase in negative affect associated with emotionally abusive practices. Performance satisfaction may influence the interpretation and experiences of psychological abuse and thus may influence the relationship between psychological abuse and mental health outcomes. Satisfaction with one's athletic performance may potentially explain some of the variation in athletes' outcomes associated with psychological abuse, however, more extensive research is needed.

The paradoxical relationship between psychological abuse and performance satisfaction may be explained by over-conformity to the sport ethic. Hughes and Coakley (30) posited the sport ethic is comprised of four tenets: self-sacrifice, complete dedication, accepting risk and playing through pain, and believing there are no limits. While over-conformity isn't inherently negative, an uncritical adherence may lead to negative consequences. In other words, if there is a belief that experiencing pain and sacrifice (e.g., experiencing maltreatment, or engaging in disordered eating behavior) is a requirement for achieving excellence in sport, negative consequences are more likely to occur. The foundation of embodying the sport ethic is entrenched with having a strong athletic identity, because overconforming “confirms and reconfirms athletic identity” (30, p. 311). Moreover, previous researchers have linked an exclusive sport identity to increased athlete satisfaction (31) and increased vulnerability to experiencing emotional abuse (32).

Several researchers have linked experiences of psychological abuse to over-conformity of the sport ethic (33, 34). For instance, McGee and colleagues (34) found a bi-directional relationship such that the sport ethic both increased athletes' vulnerability to psychological harm and psychological harm was tolerated more because of the athletes' commitment to the sport ethic. Athletes acknowledged the dominant sport ethic, specifically relating it to the predominant win-at-all-costs pressure. Athletes reported not questioning their coaches' psychological abuse, rather accepted it as part of a high-performance culture, or believing it is a necessary sacrifice for performance success. Other athletes reported being willing to accept abusive behavior in order to achieve their desired results, which increased their vulnerability to psychological abuse. Additionally, Boudreault and colleagues (35) found extreme weight control behaviors (i.e., symptoms of disordered eating) were related to experiences of weight-related maltreatment, which has been proposed as a form of psychological abuse by Willson and Kerr (9). Disordered eating may be influenced by over-conformity to the sport ethic, because athletes believe behaviors such as food restriction and excessive exercise are assets to performance and are praised by coaches (36, 37). However, there is a paucity of research correlating athlete satisfaction and the sport ethic. For instance, if an athlete is satisfied with their performance and their sport environment, they may believe it is necessary to engage in self-sacrificing or risk-taking behaviors to obtain such results. Additionally, they may feel proud of or justify their sacrifices or risks if their desired results were obtained, thus increasing their satisfaction (38). Further research is needed to explore potential links between psychological abuse, mental health outcomes, and the role of performance satisfaction.

Therefore, the purpose of this research was to examine the relationships between psychological abuse in sport, athlete satisfaction, and self-harm and eating disorder indicators, across sport types. Examining the influence of athlete satisfaction on the effects of psychological abuse is critical for informing intervention and treatment such as helping athletes understand why they may or may not be negatively impacted by experiences of psychological abuse, or why the impacts of psychological abuse may not be experienced immediately.

The first objective of this study was to investigate the relationship between psychological abuse, athlete satisfaction, eating disorder and self-harm indicators. The first hypothesis (H1) is that psychological abuse would be negatively related to athlete satisfaction, and positively related to eating disorder and self-harm indicators. The second objective was to investigate whether athlete satisfaction is a moderator in the relationship between psychological abuse and eating disorder and self-harm indicators. The second hypothesis (H2) is that athlete satisfaction would serve as a moderator or a buffer, such that athletes who experienced psychological abuse, and had high satisfaction in their sport would be less likely to experience eating disorders and self-harm. On the other hand, athletes who experienced psychological abuse and had low satisfaction in their sport would be more likely to report indicators of eating disorders and self-harm. Given the differing rates of psychological harm, eating disorders and self-harm in individual, team, and aesthetic sport athletes (22–27, 39), the third objective was to investigate whether there is a difference in the relationships between psychological abuse, athlete satisfaction, and mental health outcomes between athletes in aesthetic or weight-class sports (e.g., artistic swimming, gymnastics, wrestling) vs. non aesthetic or weight-class sports, and athletes in team sports (e.g., basketball, hockey, water polo) vs. individual sports. The third hypothesis (H3) is that the relationships between variables of interest would be stronger in aesthetic or weight-class sport athletes and in team sport athletes than their counterparts.

## 2 Methods

This study represents a component of a larger research project assessing experiences of maltreatment in a sample of Canadian National Team Athletes, including, prevalence of maltreatment, athletes' perceived impacts of maltreatment experiences on athlete satisfaction, mental health and well-being, their disclosure and reporting behaviors, and their recommendations for improving their sport experiences. Previous reported findings include the prevalence of physical, sexual, and psychological abuse, and neglect (5), and the relationships between all types of maltreatment and mental health indicators of well-being, eating disorders, and self-harm (14). The present study differs from the previously published papers in purpose, research questions, some of the measures, analyses, and findings. Contrary to the previously published papers, the inclusion criterion was only on athletes who responded to questions about psychological abuse during their tenure on the Canadian national team (*n* = 794) to explore the relationships between psychological abuse and mental health indicators, and the potential role of athlete satisfaction in influencing these relationships.

### 2.1 Participants

Participants included current and retired (maximum of 10 years retired) Canadian National Team athletes, including para and non-para athletes from any sex and from any sport with a national team. Competing on the national team was a selection criterion, as the purpose was sought to explore the experiences of athletes at the highest level of competition. Participants were required to be over the age of 16 years to participate. AthletesCAN, the association of Canadian National Team athletes, was a partner of this study and involved in the development of the survey design and facilitated the recruitment of participants. AthletesCAN is dedicated to being the collective voice for athletes, representing and advocating for the interests of athletes (40). Eligibility criteria for membership in AthletesCAN include being a current member of a Canadian National Team or retired from a National Team within the past 8 years, which aligned with our study inclusion criteria.

There were 592 (75%) current athletes and 202 (25%) retired athletes (*M*_years_ = 4.31, SD = 2.79). The sample was comprised of 496 (63%) females and 295 (47%) males (*n* = 3 did not disclose sex) national team athletes, all of whom were over 16 years of age (*M*_age_ = 27.85, SD = 9.08). The athletes in this sample were participants in 64 sports with the highest proportions of athletes represented in gymnastics, volleyball, athletics, cycling, swimming, rowing, freestyle skiing, and rugby. Participants self-reported identity characteristics including having a disability (*n* = 95, 12%), being racialized or of a racial minority (*n* = 76, 10%), LGBTQ2I+ (*n* = 59, 8%), and Indigenous (*n* = 13, 2%). There were 115 (14%) aesthetic and weight-class sport athletes and 226 (28%) team sport athletes.

### 2.2 Measures

#### 2.2.1 Psychological abuse

Psychological abuse was measured using 9 items adapted from Vertommen et al. (1) and refined by AthletesCAN. Questions included: “*you were put down, embarrassed, or humiliated*”, “*you have been criticised as a person when your performance was subpar*”, and “*you were called derogatory names or otherwise offended.*” Participants were asked to report whether each instance of psychological abuse had occurred during their time in sport (response options = Yes, No, NA). For all participants, a score representing the number of items answered “Yes” (range = 0–9) was computed and used in all analyses.

#### 2.2.2 Athlete satisfaction

Athlete Satisfaction was measured using a modified version of the Athlete Satisfaction Questionnaire (ASQ) (29), which is a comprehensive measure of the structure, processes, and outcomes of an athlete's experience. This measure accounts for perceptions of the individual and team, as well as social components of the athletes' experience, for instance, satisfaction with performance (individual and team), performance improvement, and with the coaching they received (e.g., treatment, technical style, choices in play). Some items from the original survey (29) were removed or reworded to ensure relevance. The adapted measure included 10-questions in which athletes were asked to rate their satisfaction with their sport performance, their team performance (if applicable), and their coach communication and training on a 7-point scale (1 = not at all satisfied to 7 = extremely satisfied) (29). Examples of items included: Identify the extent to which you are satisfied with, “*The verbal instructions I have received from my coach*”, “*The guidance I have received from my teammates/training partners*” and “*Reaching my performance goals.*” For all participants, a score representing the average item response was computed and used in all analyses. This scale demonstrated strong internal consistency *α* = .90.

#### 2.2.3 Eating disorder indicators

Eating disorder indicators were measured using three questions designed to gain an overall sense of athletes' history with eating disorders. Participants were asked to report whether they had: (i) thought about engaging in disordered eating behaviors (e.g., restriction, binging, purging) during their time in sport; (ii) engaged in disordered eating behaviors (e.g., restriction, binging, purging) during their time in sport; and (iii) sought treatment for disordered eating or an eating disorder (response options: Yes, No, N/A). This scale was also used in a previously published paper on the relationships between experiences of maltreatment and mental health indicators (14). Item responses were reduced to individual scores using principal axis factoring and scores for each construct were extracted using the regression method. The three eating disorder items loaded onto a single factor accounted for 88% of the variance, and the regression method was used to extract scores for each participant on an eating disorder factor score. Factor scores for eating disorders were included in all analyses as composite scores.

#### 2.2.4 Self-harm indicators

Self-harm indicators were measured using three questions designed to gain an overall sense of athletes' history with self-harm and suicidality. Participants were asked to report whether they had: (i) thought about engaging in self-harm or suicide during their time in sport; (ii) engaged in self-harm or suicide during their time in sport; and (iii) sought treatment for self-harm or suicide (response options: Yes, No, N/A). This scale was also used in a previously published paper on the relationships between experiences of maltreatment and mental health indicators (14). Item responses were reduced to individual scores using principal axis factoring. The self-harm items loaded onto a single factor accounted for 84% of the variance, and the regression method was used to extract scores for each participant on a self-harm factor. Factor scores for self-harm were included in all analyses as composite scores.

### 2.3 Procedure

Following ethical approval from the University's Ethics Review Board, AthletesCAN conducted recruitment through email and posting publicly on their social media platforms (Instagram, Twitter and Facebook). AthletesCAN maintains a database which includes athletes' contact information, which was used to distribute the recruitment email to 6,239 athletes. The recruitment email contained inclusion criteria, links to the anonymous survey, which was offered in French and English (Canada's two official languages), and a letter of information. The letter of information outlined the voluntary nature of the study as well as assurances of confidentiality and anonymity with only aggregated data presented. The survey was available to participants for 1 month, during which time, two reminder emails were sent to encourage completion. Athletes were not compensated for their participation. The survey took approximately 15 min to complete and submitted surveys were received directly by the research team.

### 2.4 Data analysis

To address the research questions, Structural Equation Modeling (SEM) analyses were conducted using MPlus version 8 (41). All variables were grand mean centered prior to being included in the model. Relevant composite scores for each variable were compiled prior to analysis. To address the first research question, two separate models with athlete satisfaction and psychological abuse as the independent variables were constructed, with self-harm and eating disorder indicators as the respective dependent variables. To address the second research question, an interaction term was created (psychological abuse × athlete satisfaction) and included in the model as an independent variable. Finally, four multigroup SEMs were conducted with aesthetic/weight-class athletes (aesthetic/weight-class = 1, non-aesthetic/weight-class = 0) and team sport athletes (team sport = 1, non-team sport = 0) as the grouping variables, and self-harm and eating disorders as outcome variables. Aesthetic athletes were classified as those who compete in appearance-oriented sports such as artistic swimming, diving, figure skating and gymnastics in which such aspects as lean, long, straight body lines influence performance outcomes. Athletes in a weight-class based sport, such as boxing or wrestling, were classified as those who require a specific weight range to be eligible to compete. Team sport athletes are those who compete in a sport in which they are on a team of three or more athletes, such as baseball, basketball, field hockey, handball, hockey, lacrosse, ringette, rugby, soccer, volleyball, and water polo.

The fit of all models was evaluated using standard criteria to determine whether the model parameters are well estimated, including a nonsignificant *χ*^2^ test, comparative-fit-index (CFI) and Tucker-Lewis-Index (TLI) >.90, root-mean-square-error-of-approximation (RMSEA) <.08 with 90% confidence intervals (CI) (42, 43). Parameters were calculated using the maximum likelihood with robust standard errors (MLR) estimator in *MPlus* 8 (41).

## 3 Results

### 3.1 Psychological abuse, athlete satisfaction and eating disorder and self-harm indicators

The proportion of athletes who reported experiencing psychological abuse was 60% (*n* = 478), eating disorder indicators was 24% (*n* = 191), and self-harm indicators was 18% (*n* = 140). Means, standard deviations and correlations for all variables are presented in Table 1. Supporting the first hypothesis (H1), psychological abuse was negatively related to athlete satisfaction and positively related to eating disorder and self-harm indicators.

**Table 1:** Means, SDs, ranges and correlations between athlete satisfaction, psychological abuse, eating disorder and self-harm indicators*.*

	Mean	SD	Range	Psych. abuse	Self-harm	Eating disorder	Athlete satisfaction
Psychological abuse	.25	.28	0–1	1	.252*	.311*	−.316*
Self-harm	.10	.24	0–1		1	.315*	−.151*
Eating disorder	.15	.28	0–1			1	−.100*
Athlete satisfaction	57.32	11.59	18–80				1

*n* = 794.

* Correlation is significant at the .01 level.

### 3.2 Athlete satisfaction as a moderator of psychological abuse on eating disorders and self-harm

**Figure 1:**
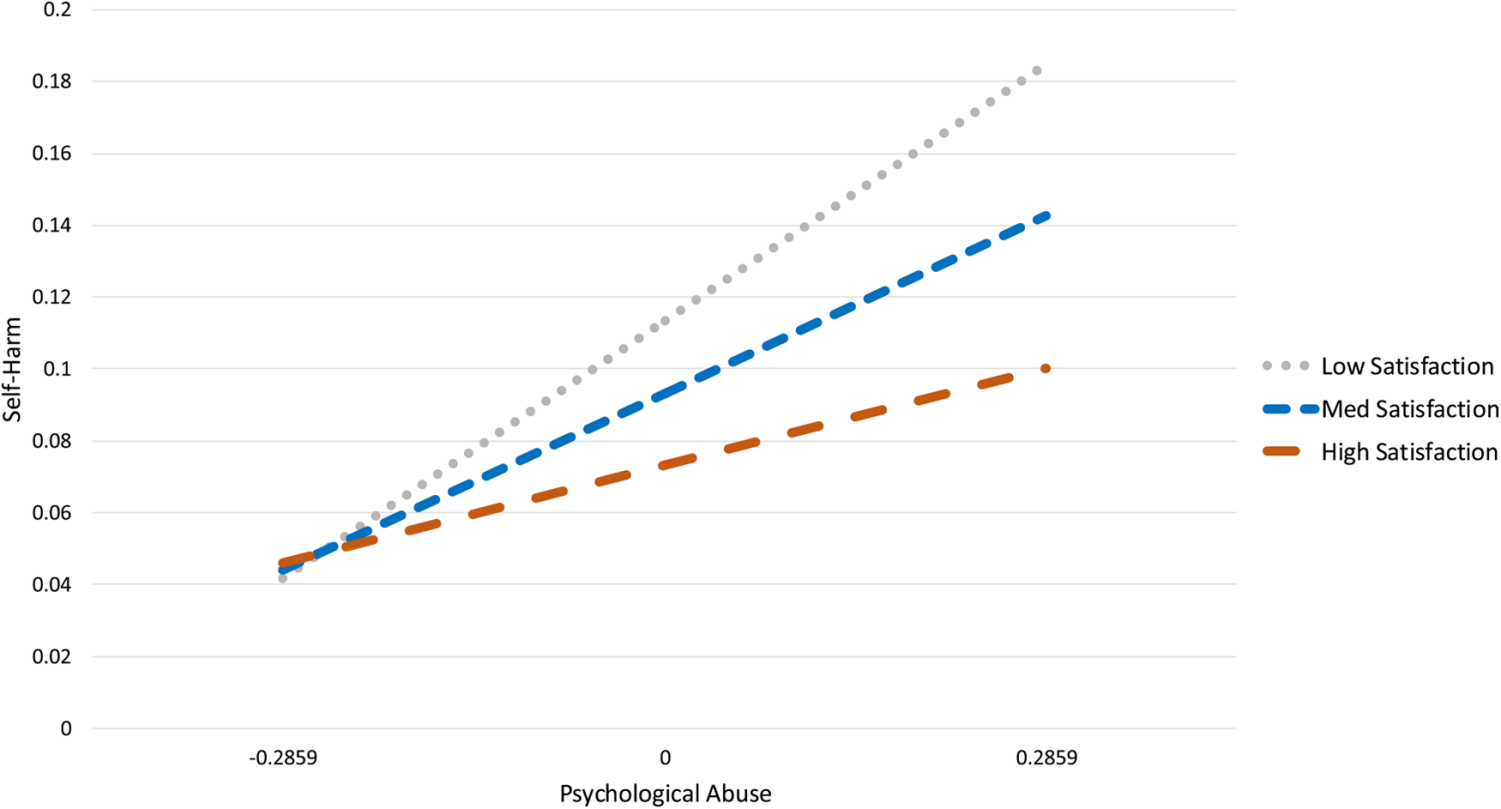
The moderating effect of athlete satisfaction on the relationship between psychological abuse and self-harm indicators.

The first model examined the effects of psychological abuse, athlete satisfaction and their interaction (psychological harm *athlete satisfaction) on self-harm indicators. With the exception of a significant chi-square test, the model fit the data well, *χ*^2^_(3)_ = 63.02, *p* = .000, CFI = 1.00, TLI = 1.00, RMSEA = .00 (90% CI = [.000, .000]). Psychological abuse was a significant predictor of self-harm indicators, while athlete satisfaction was not. The interaction between psychological abuse and athlete satisfaction was a significant predictor of self-harm indicators (Interaction = −.42, SE = .16, *p* = .01 *R*^2^ = .08). Estimates, standard errors, *p*-values, and effect sizes are presented in Table 2. In accordance with our hypothesis (H2), the plotted interaction (Figure 1) demonstrates that when athlete satisfaction is high, the effect of psychological abuse on self-harm indicators is reduced.

**Table 2:** Estimates, SE and *p*-values for eating disorder and self-harm indicators.

	Self-harm			Eating disorders		
	Estimate	S.E.	*p*-value	Estimate	S.E.	*p*-value
Psychological abuse	.66	.17	.00	−.01	.16	.94
Athlete satisfaction	.00	.05	.98	−.06	.05	.19
Interaction	−.42	.16	.01	.32	.16	.04
*R* ^2^	.08			.10		

Estimates are considered significant at *p* < .05. *n* = 792.

**Figure 2:**
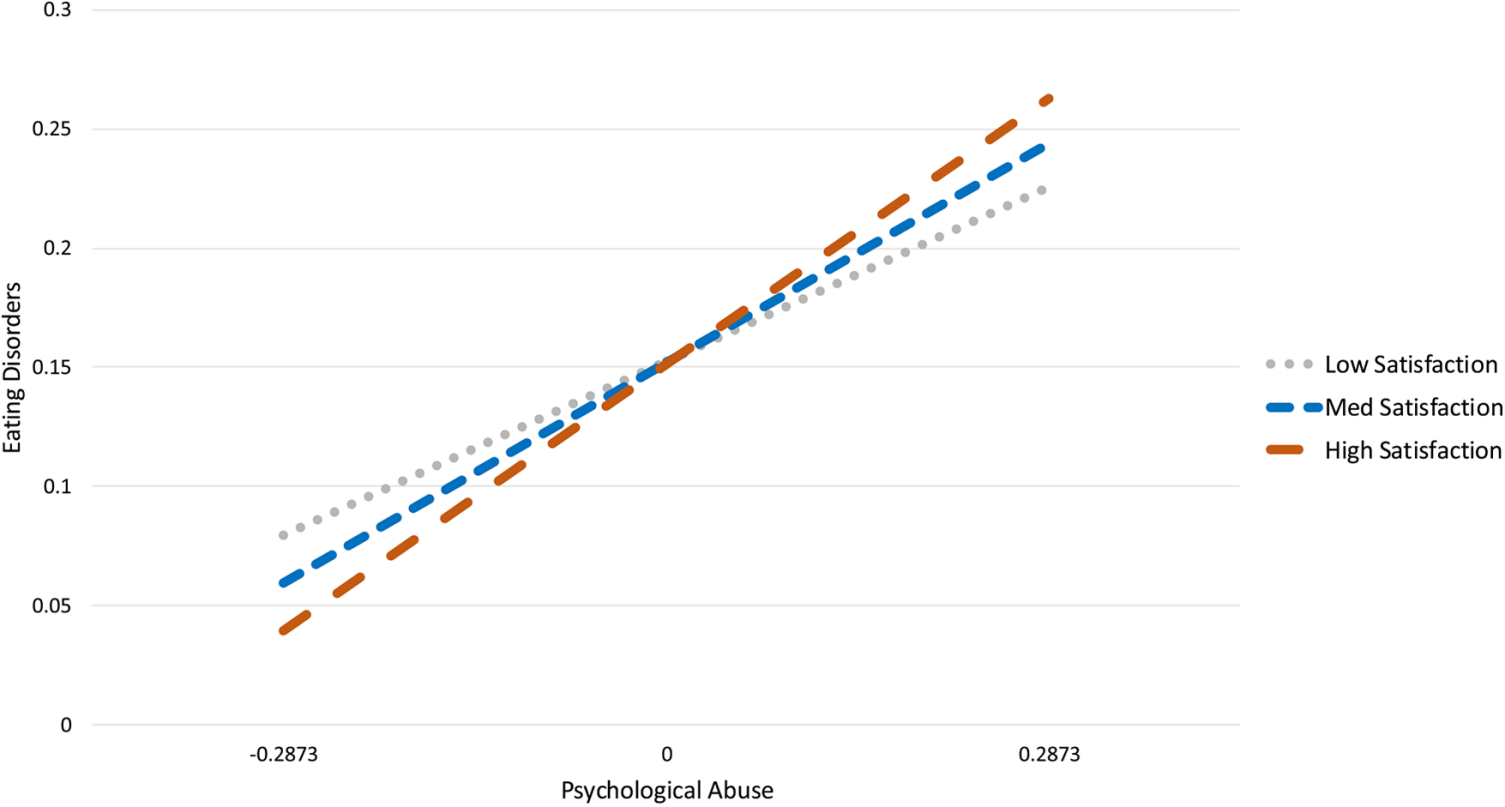
The moderating effect of athlete satisfaction on the relationship between psychological abuse and eating disorder indicators..

The second model examined the effects of psychological abuse, athlete satisfaction and their interaction (psychological abuse *athlete satisfaction) on eating disorder indicators. With the exception of a significant chi-square test, the model fit the data well, *χ*^2^_(3)_ = 85.06, *p* = .000, CFI = 1.00, TLI = 1.00, RMSEA = .00 (90% CI = [.000,.000]). Psychological abuse and athlete satisfaction were not significant predictors of eating disorder indicators, but their interaction was significant (Interaction = .32, SE = .16, *p* = .04 *R*^2^ = .10). Estimates, standard errors, and *p*-values are presented in Table 2. Contrary to our hypothesis (H2), the plotted interaction (Figure 2) demonstrates that athlete satisfaction does not buffer against the effects of psychological abuse on eating disorder indicators, as the relationship between psychological abuse and eating disorders is strengthened when athlete satisfaction is high.

### 3.3 Psychological abuse and athlete satisfaction in aesthetic/weight-class and team sport athletes

There were 105 aesthetic and weight-class athletes (13%) and 206 team sport athletes (26%). The mean of athlete satisfaction was higher in aesthetic/weight-class athletes (*M* = 58.96, SE = 1.13) than non-aesthetic/weight-class athletes (*M* = 57.00, SE = .44) and in team sport athletes (*M* = 58.71, SE = .70) than non-team sport athletes (*M* = 56.75, SE = .49). Reported self-harm was higher in aesthetic and weight-class athletes (*M* = .15, SE = .03) than non-aesthetic or weight class athletes (*M* = .09, SE = .01) and in non-team sport athletes (*M* = .11, SE = .01) than team sport athletes (*M* = .07, SE = .02). Similarly, reported eating disorder indicators were higher in aesthetic and weight-class athletes (*M* = .26, SE = .03) than non-aesthetic or weight class athletes (*M* = .13, SE = .01) and in non-team sport athletes (*M* = .16, SE = .01) than team sport athletes (*M* = .09, SE = .02).

Four multigroup models were constructed to examine the final research question of whether the interaction between psychological abuse and athlete satisfaction differs between groups of athletes who are in weight based/aesthetic sports vs. non aesthetic/weight-class (models 1 and 2) and athletes who are in team sports vs. individual sports (models 3 and 4). The first multigroup model examined aesthetic and non-aesthetic/weight-class sport athletes on self-harm indicators (Table 3). With the exception of a significant chi-square test, the model fit the data well, *χ*^2^_(6)_ = 63.02, *p* = .000, CFI = 1.00, TLI = 1.00, RMSEA = .00 (90% CI = [.000,.000]). The results demonstrated the interaction between psychological abuse and athlete satisfaction on self-harm indicators was significant only in non-aesthetic/weight-class sport athletes (Interaction = −.47, SE = .16, *p* = .00 *R*^2^ = .07). The second multigroup model examined aesthetic and non-aesthetic/weight-class sport athletes on eating disorder indicators (Table 3). With the exception of a significant chi-square test, the model fit the data well, *χ*^2^_(6)_ = 73.77, *p* = .000, CFI = 1.00, TLI = 1.00, RMSEA = .00 (90% CI = [.000,.000]). The results demonstrated that in partial accordance with our hypotheses, the interaction between psychological abuse and athlete satisfaction on eating disorder indicators was marginally significant in aesthetic/weight-class sport athletes (Interaction = .81, SE = .47, *p* = .08 *R*^2^ = .20), but not significant in non-aesthetic/weight-class sport athletes (H3).

**Table 3:** Estimates, SE and *p*-values in aesthetic and non-aesthetic/weight-based sport athletes.

	Aesthetic/weight-based			Non-aesthetic/weight-based		
	Estimate	S.E.	*p*-value	Estimate	S.E.	*p*-value
Model 1: self-harm						
Psychological abuse	.34	.52	.51	.70	.17	.00
Athlete satisfaction	−.22	.13	.10	.04	.05	.48
Interaction	−.14	.50	.78	−.47	.16	.00
*R* ^2^	.13			.07		
Model 2: eating disorders						
Psychological abuse	−.42	.49	.39	.08	.17	.63
Athlete satisfaction	−.19	.13	.13	−.05	.05	.34
Interaction	.81	.47	.08	.17	.16	.29
*R* ^2^	.2			.07		

Estimates are considered significant at *p* < .05. *n* = 792.

The third multigroup model examined team and non-team sport athletes on self-harm indicators (Table 4). With the exception of a significant chi-square test, the model fit the data well, *χ*^2^_(6)_ = 64.18, *p* = .000, CFI = 1.00, TLI = 1.00, RMSEA = .00 (90% CI = [.000,.000]). The results demonstrated the interaction between psychological abuse and athlete satisfaction on self-harm indicators was significant only in non-team sport athletes (Interaction = −.54, SE = .18, *p* = .00 *R*^2^ = .09). The fourth multigroup model examined team and non-team sport athletes on eating disorder indicators (Table 4). With the exception of a significant chi-square test, the model fit the data well, *χ*^2^_(6)_ = 86.36, *p* = .000, CFI = 1.00, TLI = 1.00, RMSEA = .00 (90% CI = [.000,.000]). The results demonstrated that contrary to our hypotheses, the interaction between psychological abuse and athlete satisfaction on eating disorder indicators was not significant in both team and non-team sport athletes (H3).

**Table 4:** Estimates, SE and *p*-values in team and non-team sport athletes.

	Team sport			Non-team sport		
	Estimate	S.E.	*p*-value	Estimate	S.E.	*p*-value
Model 1: self-harm						
Psychological abuse	.04	.36	.91	.78	.19	.00
Athlete satisfaction	−.02	.09	.82	.01	.06	.85
Interaction	.18	.34	.61	−.54	.18	.00
*R* ^2^	.05			.09		
Model 2: eating disorders						
Psychological abuse	−.14	.34	.67	.00	.19	1.00
Athlete satisfaction	−.07	.09	.44	−.06	.06	.30
Interaction	.50	.32	.12	.29	.18	.10
*R* ^2^	.14			.09		

Estimates are considered significant at *p* < .05. *n* = 792.

## 4 Discussion

The current study contributes to existing literature by exploring the relationships between psychological abuse, athlete satisfaction, eating disorders and self-harm indicators. Experiencing psychological abuse was negatively related to athlete satisfaction, and positively related to self-harm and eating disorder indicators. The findings are unique in showing the potential buffering effect of athletes' satisfaction on eating disorders and self-harm indicators. Building on Vertommen and colleagues’ (39) findings of increased vulnerability of non-team sport athletes to psychological harm, team sport and non-weight-based sport athletes in the current study reported lower self-harm and eating disorder indicators compared to non-team sport athletes and weight-based sport athletes. Additionally, team sport athletes reported higher satisfaction. Further, results indicated that for athletes who have high satisfaction with their sport, the relationship between psychological abuse and self-harm is weakened, which suggests a potential buffering effect. The inverse effect was found for eating disorders, such that for athletes with high satisfaction, the relationship between psychological abuse and eating disorders was strengthened, specifically in aesthetic/weight-class sport athletes. Conversely, for athletes in non-aesthetic/weight-class sports and team sports, athlete satisfaction buffered the effects of psychological abuse on self-harm indicators.

While the present study indicated a negative relationship between psychological abuse and athlete satisfaction, previous studies have found conflicting results. For instance, Bekiari and Syrmpas (44) reported experiencing verbal aggression (e.g., insults, negative judgements) from coaches was significantly negatively related to athlete satisfaction and performance. In particular, verbal aggression emerged as an important negative predictor of athlete satisfaction. McGee and colleagues (34) explored the links between psychological abuse and over-conformity to the sport ethic, which illuminated the complexity of athletes' relationship to their belief in the functionality of psychological abuse. Athletes reported that psychologically abusive behaviors were discouraging, decreased enjoyment, and increased their desire to withdraw from sport, responses that reportedly became more salient over time. The present study's findings are congruent with athletes reporting less satisfaction when experiencing psychological abuse. On the other hand, the athletes in McGee and colleagues' (34) study reported psychological abuse contributed to higher effort, such that athletes were motivated to push harder, or sacrifice external commitments to avoid being yelled at by their coaches (34). This notion is consistent with previous qualitative studies on psychological abuse in which athletes have reported positive impacts on sport performance satisfaction (12, 28, 45).

The narrative of psychological abuse having a positive impact on performance has been perpetuated in sport by several stakeholders, including coaches, parents, and sport administrators (46). For instance, athletes have justified their coaches’ negative behaviors as necessary because “if a coach is too lenient, that just doesn't get results” (28, p. 97) or are effective means of encouragement: “… they were just trying to get you motivated” (47, p. 131). Similarly, some coaches justify harmful behaviors as being in the best interest of the athlete: “I worked her really hard and really pushed her. She rose four places in the rankings and could go to international competitions. That is in a child's best interest” (46, p. 137). More research is needed to understand the links between athlete satisfaction and performance success; however, the findings from the present study add to this literature by demonstrating psychologically abusive behaviors can have a negative impact on athlete satisfaction. Additionally, the negative relationship between psychological abuse and athlete satisfaction challenges the belief of psychologically abusive behaviors being necessary for performance and instead, may *hinder* performance satisfaction.

The negative impact of psychological abuse on self-harm indicators is consistent with previous literature in sport and general child abuse (12, 48–50), but the findings from the present study provide additional insight into the complexity of the relationships, namely athlete satisfaction can impact experiences of self-harm. Stirling and Kerr (28) identified athletes' responses to psychological abuse can differ over time, such that the same athlete may have a different response to the same coaching behaviors throughout their career. Additionally, the extent of athletes' reactions to psychological abuse was related to their satisfaction with performance (28), which is consistent with our current findings, namely athlete satisfaction provided a buffer between the effects of psychological abuse on self-harm. Specifically, athlete satisfaction weakened the relationship between psychological abuse and self-harm. Moreover, the present findings build on understanding the nuance between sport categories, such that self-harm can be buffered for non-team-sport athletes by athlete satisfaction. Previously, researchers have demonstrated non-team sport athletes are more likely to suffer from mental health disorders and self-harm indicators (26, 51, 52), as such, athlete satisfaction may be a protective factor in the outcomes, particularly for this subset of athletes. One interpretation of this finding is that the effects of abuse may be masked by one's perception of performance as satisfactory. Consistent with previous studies linking the over-conformity of the sport ethic to psychological abuse, when success has been achieved by the athlete, the methods used to achieve the success are not questioned or are justified by the athlete (33, 34). As such, the effects of harmful practices may not have negative effects in the moment but this does not mean they do not or will not be experienced later (34). Although psychological abuse has been reportedly normalised in sport (9, 50), athletes have reported not realizing the extent of its negative impacts until they left their sport (12, 47). For instance, one retired athlete reflected “I discovered a lot of things with therapy about how I was treated… I wasn't aware that like, that's not how a coach is actually supposed to treat you” (12, p. 85). Therefore, we urge practitioners to be cautious of engaging in psychologically harmful behaviors because the effects of these behaviors can be masked by successful performance outcomes. More research is needed to understand why these behaviors continue to be normalised and justified as a tool for performance and how to change these beliefs.

In contrast, the relationship between psychological abuse and eating disorders was strengthened by athlete satisfaction, particularly in athletes in aesthetic or weight-class sports, meaning satisfaction had a catalyzing rather than a buffering effect. The perceived connections between appearance and performance, or the “thin-to-win” discourse existing in aesthetic sport (53–56) may explain these findings. The “thin to win” discourse posits that a leaner body will produce a superior performance (57–59), a view that can be further attributed to the over-conformity to the sport ethic. Papathomas (36) suggested engaging in disordered eating behaviors including abstaining from bad foods and tolerating persistent hunger uphold the values of the sport ethic. This author posited athletes are often pressured into engaging in disordered eating behaviors because of the belief they are necessary for performance. In particular, athletes feel they “must engage in disordered eating or risk being considered not committed enough or not tough enough… athletes in effect have a choice between becoming mentally ill or appearing mentally weak; damned if they do and damned if they don't” (2015, p. 105). In relation to the present study, it is possible the desire to achieve high performance can drive athletes to engage in eating disorder behaviors because of the belief they enhance performance outcomes. Eating disorders are multifaceted and can be influenced by a multitude of external and internal factors (58). Individual risk factors can include genetics, age, self-esteem, and personality traits (e.g., perfectionism), whereas external risk factors can include body shaming and pressure on appearance, trauma, and coaching behaviors (53) Additionally, there continues to be a win-at-all costs mentality in sport, in which performance is valued over all else. The interconnected nature of eating disorders, therefore, could be exacerbated by experiencing psychologically harmful behaviors in sport and pressures to perform. For instance, experiences of psychological abuse (being screamed at, told you're not good enough), combined with an internalised thin-to-win mentality, in a culture that values performance and self-sacrifice can create a volatile condition in which eating disorders can thrive. Aesthetic sports, in which scoring systems have appearance criteria embedded in them, may further exacerbate the problem (59). This could also explain the catalyzing effect of athlete satisfaction in weight-based sports, because athletes were more willing to self-sacrifice by way of disordered eating to achieve desired sport outcomes. Furthermore, athletes may have been more satisfied with their experience because of their belief self-sacrifice was necessary for performance attainment. The relationships between psychological abuse and eating disorders (12, 48, 60) and between aesthetic sports and eating disorders (23, 61), the drive for thinness for performance and eating disorders (23) have been studied independently, but not collectively. The findings from the present study contribute to the literature by exploring the relationship between multiple facets and are unique because it explored the relationship between these factors and athlete satisfaction. From a practitioner's perspective, more attention is needed to address the underlying factors (i.e., maltreatment) that continue to have negative impacts on mental health and performance. Given the present findings, athletes in weight-based sports who have increased vulnerability deserve particular attention. A narrative shift is required to dispel the belief that psychologically abusive coaching styles and engaging in disordered eating behaviors are the most effective way to increase performance results.

Overall, the findings from this study demonstrate the salient influence of athlete satisfaction on the experiences of psychological harm and its outcomes. The results of this study can be partially explained by the over-conformity to the sport ethic, because athletes are expected to demonstrate commitment to their sport through self-sacrifice, dedication, and playing through pain (30). Athletes may also be more satisfied with their experience if they have overcome pain and suffering, as it reconfirms their athletic identity and pursuit of distinction (30, 31, 62). In other words, if an athlete has self-sacrificed, they may be more satisfied with their results. This is seemingly apparent in the higher rates of athlete satisfaction in athletes of weight-based sports because athletes may self-sacrifice through disordered eating behaviors and become more satisfied as a result. The sport ethic could also partially explain the differences in the mental health outcomes of eating disorders and self-harm. Disordered eating behaviors can be viewed as facilitative of a performance outcome, thus experienced at higher levels, whereas self-harm is more destructive to performance, and thus may be under-reported. Despite these explanations, it is imperative to recognize these behaviors are potentially harmful to athletes' health and well-being and, therefore, should be actively addressed and prevented.

## 5 Limitations and future directions

This study provided exploratory insights into the relationships between psychological abuse, athlete satisfaction, and two mental health indicators of self-harm and eating disorders. Given this was part of a larger project assessing maltreatment in sport, broad measures of mental health indicators were used. As such, the current methods were limited by the use of non-validated questionnaires, subsets of validated scales, and the use of a narrow population of athletes (i.e., national team athletes). Future studies could look at these relationships in more in-depth, including the use of psychometrically validated scales and a broader list of mental health indicators (e.g., anxiety, depression, well-being). While the present study looked at categories of sports as risk factors of eating disorders and self-harm, many other risk factors can contribute to mental health challenges were not considered, and it is important to consider the interplay between risk factors. Finally, findings did not differentiate between maltreatment perpetrated by coaches, teammates, parents, or other perpetrators. Future research could assess the outcomes of psychological abuse as it pertains to the various perpetrators of harm.

From an applied perspective, findings in this study indicate the associations between psychological abuse and negative mental health indicators. Given the high prevalence of psychological abuse (1–5), more work is needed to prevent and address psychological abuse in sport. Despite the growing body of evidence indicating the scope and negative outcomes of psychological abuse, it continues to be the most frequently reported form of harm. Future research could address the research-to-practice gap to cease this normalized practice.

## 6 Conclusion

The high prevalence of psychological abuse in sport is becoming increasingly reported in research and practice (1–5). This study contributes to emerging literature by demonstrating the detrimental effects of psychological abuse on athletes, specifically with respect to the increased risk of eating disorder and self-harm indicators. Additionally, findings from this study demonstrate psychological abuse can negatively impact athlete satisfaction, and athlete satisfaction can both buffer and catalyze the effects of harm. Together, results indicate the need to prevent and address psychologically harmful practices in sport. Recommendations include increasing education for coaches and other sport participants, particularly on the scope of what behaviors constitute psychological abuse and the potentially harmful impacts of these behaviors on athletes. Increased awareness of the dangers of promoting adherence to the sport ethic, which emphasizes a culture that promotes self-sacrifice and accepting excessive demands, is also necessary given the potential negative impacts on athlete mental health. Stronger screening policies to uncover mental health challenges are needed. For example, athletes disclosing eating disorder and self-harm behaviors could be screened for experiences of maltreatment and provided appropriate psychological support (e.g., referral to a psychologist/psychiatrist). Interventions in coaching, including enforcing consequences (e.g., sanctions, removal of position) and education of appropriate coaching styles may also be needed. Overall, addressing the prevailing issue of psychological abuse is critical given its demonstrated negative impacts on athletes. Finally, from a mental health practitioner perspective, this study highlights the need for attention towards athletes in weight-based and individual sports given their increased risks of eating disorders and self-harm.

## Data Availability

The datasets presented in this article are not readily available because of the nature of this study, particularly the sensitive nature of maltreatment, participants of this study did not give written consent for their data to be shared publicly. Therefore, the supporting data is not available. Requests to access the datasets should be directed to erin.willson@utoronto.ca.
